# Supplementary material for: Assessing the extent and timing of chemosensory impairments during COVID-19 pandemic
Source: Sci Rep. 2021 Sep 1;11:17504. doi: 10.1038/s41598-021-96987-0 (PMC8410776; doi:10.1038/s41598-021-96987-0)
Supplement: Supplementary file 1 — Supplementary Information. [file 41598_2021_96987_MOESM1_ESM.pdf]

Supplementary material

Assessing the extent and timing of chemosensory impairments during COVID-19 pandemic

Cinzia Cecchetto[1], Antonella Di Pizio[2], Federica Genovese[3], Orietta Calcinoni[4], Alberto Macchi[5], Andreas Dunkel[2], Kathrin Ohla[6], Sara Spinelli[7], Michael C. Farruggia[8], Paule V. Joseph[9,10,11], Anna Menini[12], Elena Cantone[13], Caterina Dinnella[7], Maria Paola Cecchini[14], Anna D'Errico[15], Carla Mucignat-Caretta[16], Valentina Parma[3,17], Michele Dibattista[18]

[1]Department of General Psychology, University of Padova, Italy; [2]Leibniz-Institute for Food Systems Biology at the Technical University of Munich, Germany; [3]Department of Physiology, Monell Chemical Senses Center, USA; [4]Private practice VMPCT, Milan, Italy; [5]ENT department, Italian Academy Of Rhinology - ASST sette laghi Varese; [6] Helmut-Schmidt-University/University of the Federal Armed Forces Hamburg, Experimental Psychology Unit, Hamburg, Germany; [7]Department of Agriculture, Food, Environment and Forestry (DAGRI), University of Florence, Italy; [8]Interdepartmental Neuroscience Program, Yale University, USA;[9]National Institutes of Nursing Research, [10]National Institute of Alcohol Abuse and Alcoholism, [11]National Institutes of Health, [12]Neurobiology Section, SISSA, International School for Advanced Studies, Italy;[13]Department of Neuroscience, ENT section, Federico II University of Naples, Italy; [14]Department of Neurosciences, Biomedicine and Movement Sciences, Anatomy and Histology Section, University of Verona, Italy; [15]Department of Neurobiology, Goethe Universität Frankfurt, Germany;[16]Department of Molecular Medicine, University of Padova, Italy; [17]Department of Psychology, Temple University, USA; [18]Department of Basic Medical Sciences, University of Bari A. Moro

Corresponding Author:

Michele Dibattista, PhD

Department of Basic Medical Sciences,

Neuroscience and Sense Organs,

University of Bari A. Moro,

Piazza Giulio Cesare n.11, 70125 Bari, Italy.

e-mail: michele.dibattista@uniba.it

Table 1s. Frequency table of the pattern of chemosensory loss and recovery clusters in relation to days between the date of onset and completion of the questionnaire. Chemosensory loss clusters: 1 = moderate smell/taste loss and preserved chemesthesis; 2 = substantial smell, taste, and chemesthesis loss; 3 = substantial smell and taste loss, but preserved chemesthesis.

**Chemosensory loss**	**Time intervals**	**Chemosensory recovery**	**Frequency**
1	<30 days	Partial	30
1	<30 days	Substantial	5
1	>90 days	Partial	5
1	31 - 60 days	Partial	73
1	31 - 60 days	Substantial	10
1	61 - 90 days	Partial	9
2	<30 days	Partial	45
2	<30 days	Substantial	67
2	>90 days	Partial	6
2	>90 days	Substantial	5
2	31 - 60 days	Partial	117
2	31 - 60 days	Substantial	256
2	61 - 90 days	Partial	8
2	61 - 90 days	Substantial	12
3	<30 days	Partial	26
3	<30 days	Substantial	27
3	>90 days	Partial	11
3	>90 days	Substantial	1
3	31 - 60 days	Partial	127
3	31 - 60 days	Substantial	113
3	61 - 90 days	Partial	14
3	61 - 90 days	Substantial	7

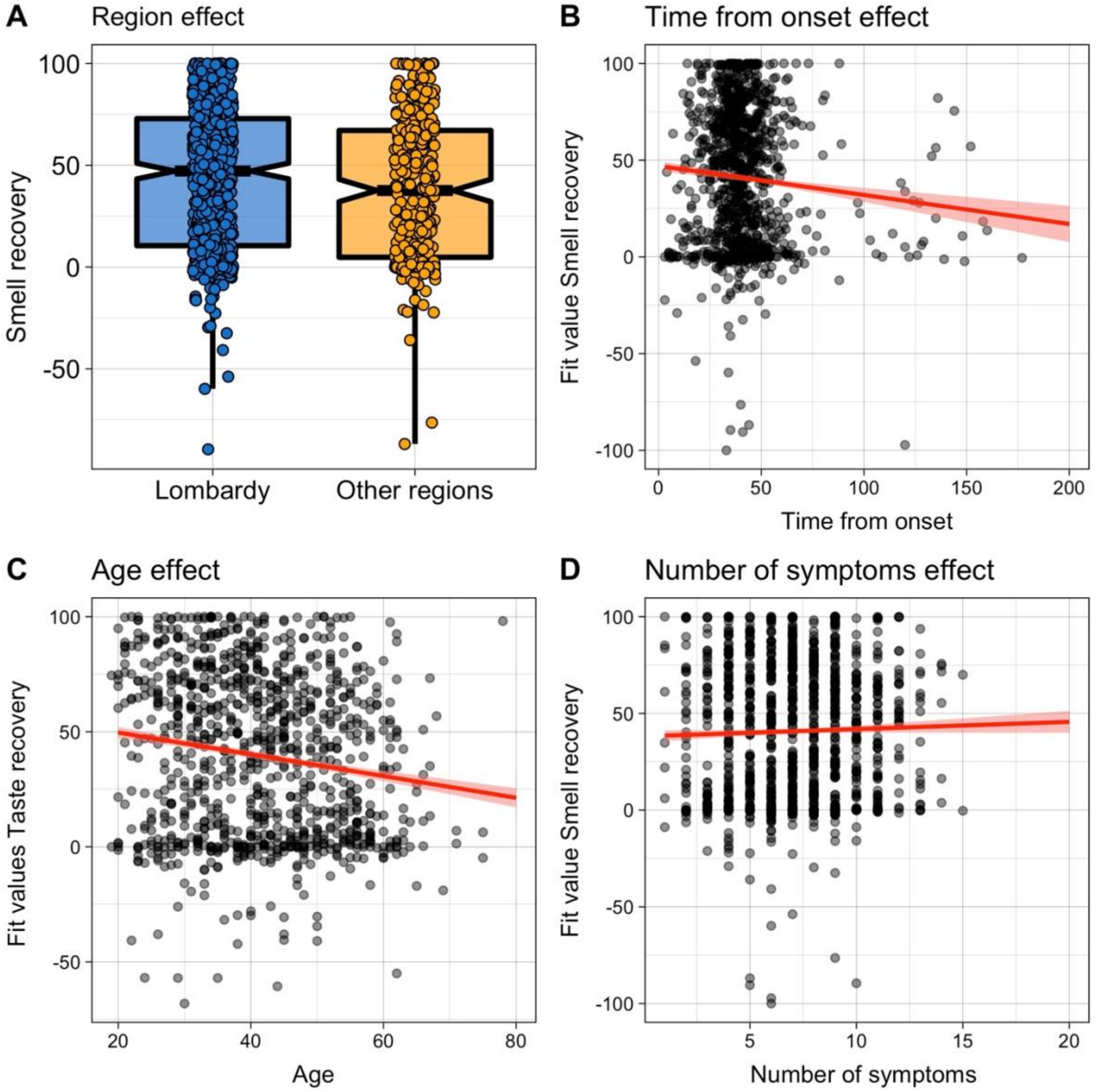

Figure 1s. Significant main effect predictors of smell recovery. A) Data distribution by region. Boxplots depict the median (horizontal black line) and quartile ranges of the distribution, whiskers indicate maximum and minimum values, colored dots represent data distribution. B) C), and D) Main effect of time from the onset, age, and number of the symptoms, with data distribution as black and grey dots and fitted lines in red.

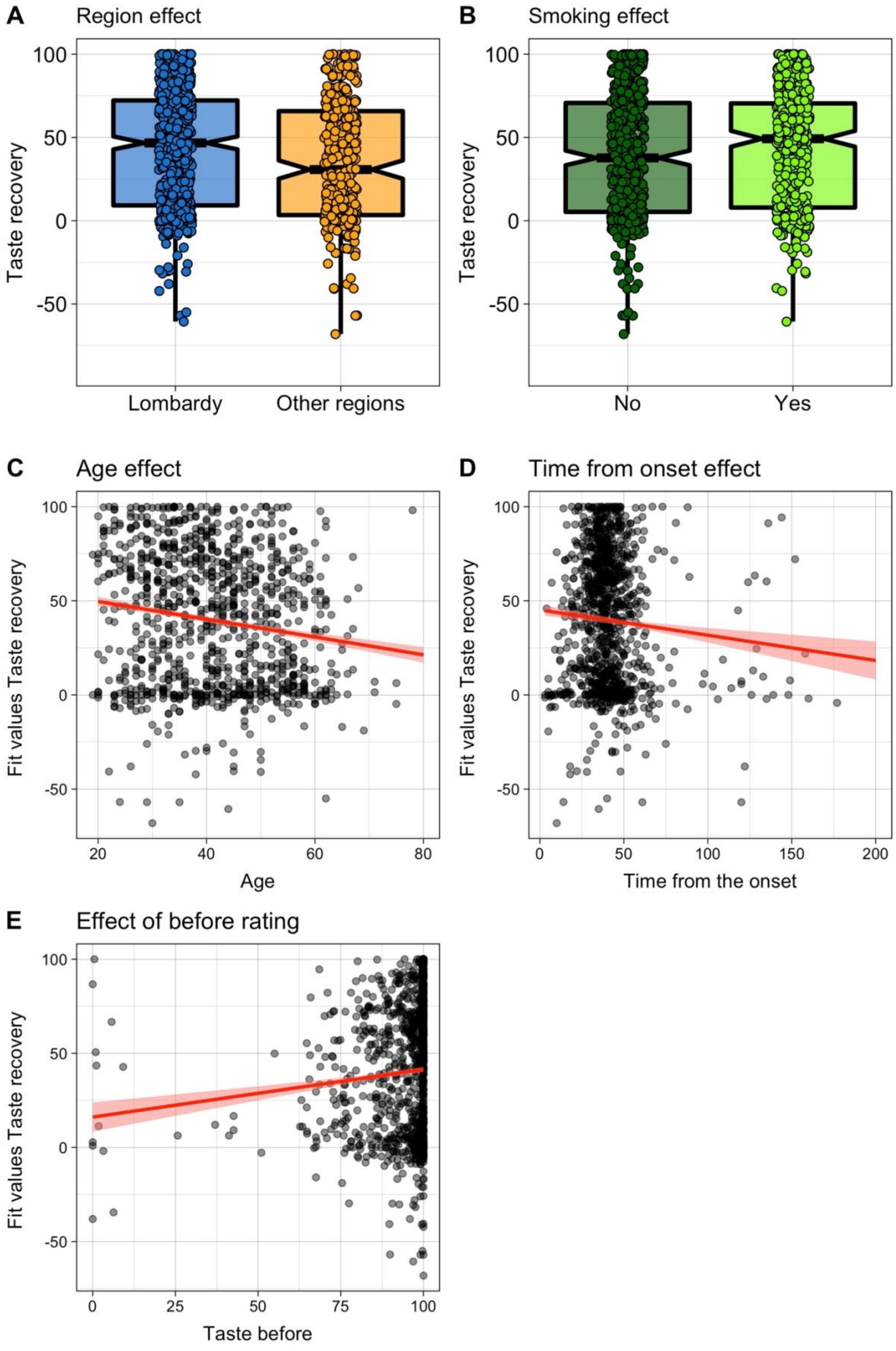

A
Region effect
Taste recovery
Lombardy
Other regions
B
Smoking effect
Taste recovery
No
Yes
C
Age effect
Fit values Taste recovery
Age
D
Time from onset effect
Fit values Taste recovery
Time from the onset
E
Effect of before rating
Fit values Taste recovery
Taste before

Figure 2s. Significant main effect predictors of taste recovery. A) and B) Data distribution by region and by smoking. Boxplots depict the median (horizontal black line) and quartile ranges of the distribution, whiskers indicate maximum and minimum values, colored dots represent data distribution. C), D), E) Main effect of age, time from onset, and before rating, with data distribution as black and grey dots and fitted lines in red.

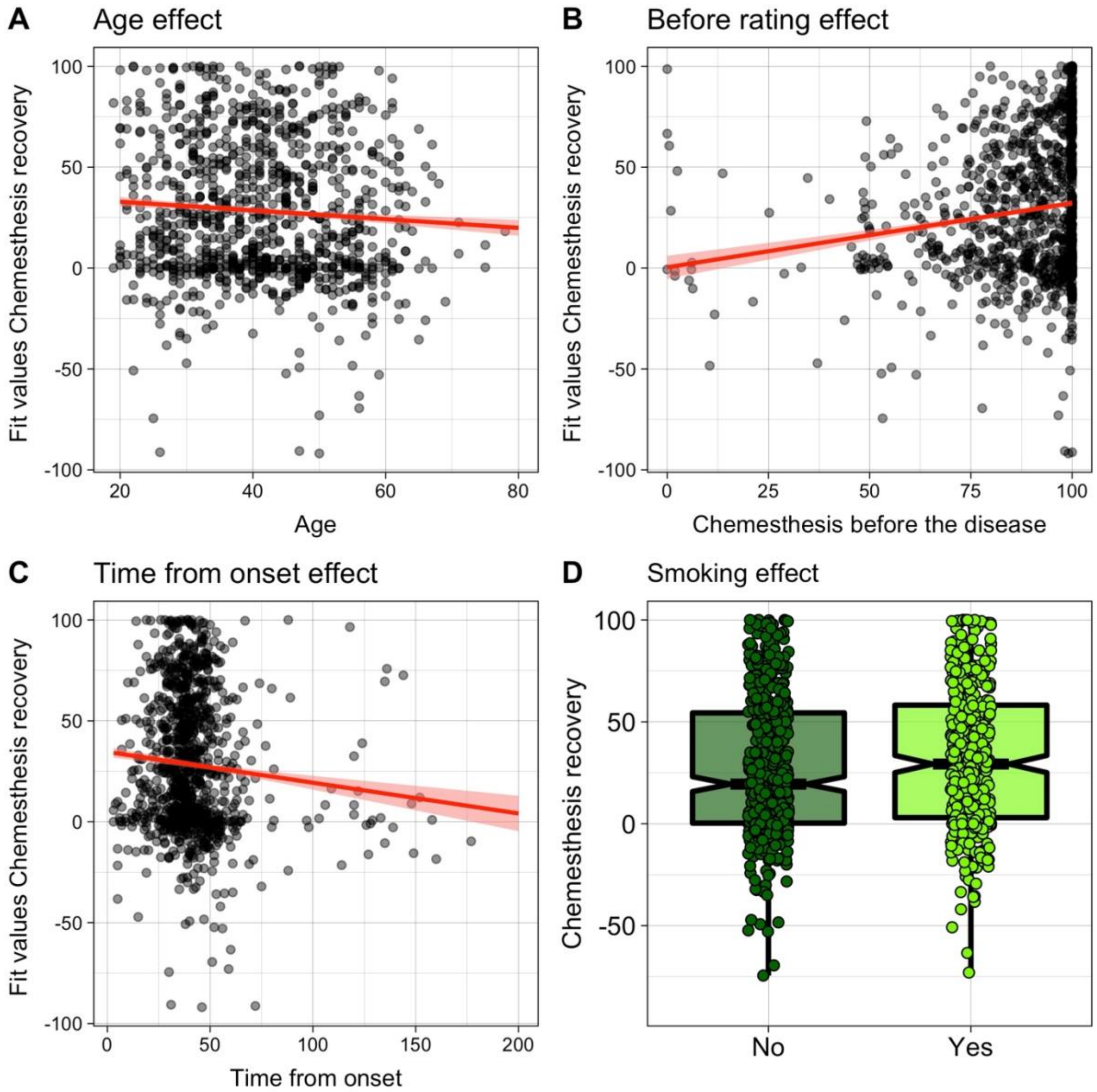

Figure 3s. Significant main effect predictors of chemesthesis recovery. A), B), and C) Main effect of age, before rating, and time from the onset of the disease with data distribution as black and grey dots and fitted lines in red. D) Data distribution by smoking. Boxplots depict the median (horizontal black line) and quartile ranges of the distribution, whiskers indicate maximum and minimum values, colored dots represent data distribution.
